# Multidimensional Property Supplementation: A Method for Discovering and Describing Emergent Qualities of Concepts in Grounded Theory Research

**DOI:** 10.1177/1049732320970488

**Published:** 2020-12-17

**Authors:** Linus Johnsson

**Affiliations:** 1 Uppsala University, Uppsala, Sweden

**Keywords:** grounded theory, theory development, methodology, epistemology, concepts, qualitative, Sweden

## Abstract

Multidimensional property supplementation is a grounded theory method for analysis that conceives of concepts as multidimensional spaces of possibilities. It is applied in an iterative process comprising four steps: *expansion*, whereby vague codes are split and contraries postulated; *abstraction* of practically significant differences in terms of properties and dimensions; *geometrization* of properties to create conceptual subspaces that supplant subcategories and have additional, emergent qualities; and *unification* of the concept by validating it against data and relieving it of properties that do not tie in sufficiently with other concepts. Multidimensional conceptual models encourage the researcher to elaborate properties that explain, predict, or guide action. Fully developed, they can be easily connected to others in a process and function, by virtue of their emergent qualities, as falsifiable hypotheses in their own right. For these reasons, multidimensional property supplementation is open to epistemological justification without presuming acceptance of techniques specific to grounded theory.

## Introduction

Grounded theory, a research methodology with roots in pragmatism and symbolic interactionism (Annells, 1996; Robrecht, 1995), is often associated with purely qualitative research (Charmaz, 2017, p. 2), although it was originally intended for both qualitative and quantitative purposes (Glaser, 1999, p. 842). Applying grounded theory methods correctly while maintaining openness and theoretical sensitivity presents the researcher with significant challenges. The merits and perils of *substantive* preconceptions have been discussed extensively (Becker, 1993; Heath & Cowley, 2004; Kelle, 2007; Kools et al., 1996; Sandelowski, 2010; Thorne, 2011; Walker & Myrick, 2006; Wuest, 2000). There have, in contrast, been far fewer efforts to problematize the presumed *formal* structure of grounded theory concepts. While emphasizing theory development (Rieger, 2019), the category formation process has been identified as the weakest part of grounded theory methodology because specific rules of inductive inference that would justify its procedures for category selection and saturation are lacking (Miller & Fredericks, 1999, p. 549).

Grounded theory concepts have been likened to puzzle pieces which, when brought together, form a complete theory (Morse, 2004, p. 1392). Before the researcher can begin developing theory that “fits and works to explain a process, and is understandable to those involved in the process” (Levers, 2013, p. 1), ideas emerging from the data must be conceptualized. At least within the Straussian tradition, this entails subsuming codes under subcategories, and subcategories under categories (Corbin & Strauss, 2008, p. 159; Morse, 2004, p. 1390), thus producing a hierarchy that emphasizes “vertical” relationships—those that connect different levels of abstraction—over the more interesting “horizontal” ones of temporality and causality. Computer-assisted qualitative data analysis software (CAQDAS) tools all but enforce this practice (Bringer et al., 2006, p. 252; Hutchison et al., 2010, p. 290) which, the strengths of grounded theory notwithstanding, may be epistemologically problematic. If it could be argued that how concepts are represented not merely *reflects* the researcher’s theoretical understanding but also *shapes* it, we might see fit to explore other possibilities.

This article describes *multidimensional property supplementation* (MPS), a method for analyzing and elaborating grounded theory concepts. The article is structured as follows. First, I point out some weaknesses of conceptual hierarchies: arbitrary representations of kinship, inconsistent interpretations of properties, and scaling issues. Second, I draw out the implications of Corbin and Strauss’s (2008) view that *similarities and differences between phenomena should be expressible in terms of properties and dimensions of concepts*, advancing its logical conclusion that concepts can be conceived of as *multidimensional spaces of possibilities*. Third, I demonstrate how MPS can be integrated into the grounded theory workflow. In essence, it is an iterative method through which properties and codes are supplemented until intuitively different events can be theoretically separated into subspaces, each of which is constituted by a unique combination of dimensions. To be selected into a multidimensional model, a property must be *practically significant*, that is, make a difference elsewhere in the theory. For this reason, MPS works best once the emerging theory contains a few other concepts to which the focal one can be related. Once a multidimensional concept has been fully developed, it can be easily connected to others in a process. I hope to show throughout that MPS rests on a solid pragmatist foundation and is theoretically rigorous, attentive to data, and conducive of reproducible results.

## Some Weaknesses of Conceptual Hierarchies

I begin by arguing that although organizing one’s categories hierarchically makes at least some sense early in the study when vertical relationships are salient, such a structure can become an impediment to later thought. My argument targets specifically the uneasy role of the *subcategory* as a bridge between the abstract (the concept) and the concrete (the codes and incidents). My critique echoes in part the Glaserian one of “labelling and then grouping” as being unnecessary (Glaser, 1992, p. 43) and potentially inhibiting (Melia, 1996, p. 376).

While concepts arise through theoretical interest and thought, or even “shuffling and playing around” with words (Layder, 1998, p. 31), subcategories emerge when diversity prompts further categorization. From a pragmatist standpoint, a subcategory earns its place in the theory by capturing a difference which is *practically significant.* To quote James (1995),

There can be no difference anywhere that doesn’t make a difference elsewhere—no difference in abstract truth that doesn’t express itself in a difference in concrete fact and in conduct consequent upon that fact, imposed on somebody, somehow, somewhere and somewhen. (p. 20)

Practically significant differences are those that *ought to* matter to the theory as a whole. To make them *actually* matter (in the sense of shaping theory), they must be theorized in terms of properties—“Characteristics that define and describe concepts”—and dimensions, which are the “Variations within properties that give specificity and range to concepts” (Corbin & Strauss, 2008, p. 159). On this account, a property represents the possibility of some particular kind of variation among the phenomena that instantiate the declaring concept, whereas a dimension stands for the realization of a specific feature along this range of possibilities. Whenever a property is discovered that theoretically distinguishes a pair of subcategories within the declaring concept, that property can be thought of as a question which is posed by their common ancestor and which they are bound to answer differently through dimension attribution. Pending such reduction, subcategories must be regarded as tentative, useful perhaps to guide further inquiry, but too vague to be of much theoretical use. Although there is nothing incoherent about capturing differences first through subcategories and only later through properties, the latter arguably makes the former redundant.

More importantly, conceptual hierarchies are problematic because they obfuscate similarities and differences between subcategories. To demonstrate, I will discuss below two principally different ways that a complex concept might be structured hierarchically. It shall be made evident that each of the alternatives, which I opt to call *deep* and *wide*, comes with its own set of problems. (On a side note, these terms are not contradictory; while a hierarchy can be made deep to avoid it becoming wide and vice versa, it is perfectly possible to end up with a hierarchy which is both deep and wide.) To illustrate what they entail, I introduce here a concept from an emerging theory (Johnsson & Nordgren, 2019) on the ethical decision making of general practitioners (GPs).

The concept in question, which I named the *voice of the self*, represents the concerns that GPs had regarding their personal survival and thriving in their working environment. It is practically significant because perceived threats to the self potentially influenced their ethical decisions. For simplicity, I present only four exemplifying codes: *return the initiative*, representing the wish to eschew responsibility for how others choose to lead their lives; *ensure consensus*, indicating the GP’s drive to maintain a cordial relationship; *reduce the agenda*, the realization that demands must be negotiated because of lack of time; and *avoid lengthy debates*, the insight that communication-wise, “less is more” if one hopes to bring the encounter to a close. Although all four codes can be seen as indicators (Layder, 1998, p. 84; Soulliere et al., 2001, p. 260) of the *voice of the self*, they differ in significant respects. As we shall see, they can be distinguished by postulating two properties, *recognition* and *cognition*, each of which is answered by two dimensions.

### Deep Hierarchies

**Figure 1.**
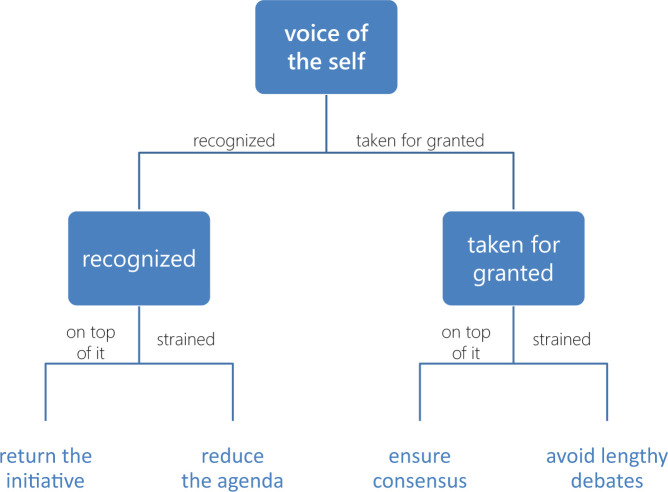
Four codes related to the *voice of the self*, visualized in a deep hierarchy that distinguishes them through two dichotomizing properties: *recognition* (with dimensions *recognized* and *taken for granted*) and *cognition* (with dimensions *on top of it* and *strained*). *Note.* Deep hierarchies distort likeness relationships, make for inconsistent interpretations of properties, and bias the researcher toward some distinctions over others.

Whenever some property remains unanswered by a subcategory, it becomes *inherited*, thus forming the basis for further categorization. The result is a *deep* hierarchy (see Figure 1). Deep hierarchies are problematic for at least three reasons.

First, the structure appears to imply that siblings are more alike than “cousins” on each level of the tree, but this does not necessarily hold. *Return the initiative* would indeed be more akin to *reduce the agenda* than to *ensure consensus* if the property *recognition* were more practically significant than *cognition.* In the event that both properties are equally practically significant, the shape of the tree will be arbitrary and misleading.

Second, because each step down the hierarchy introduces conceptual assumptions, inherited properties (in our example, *cognition*) might be interpreted differently in different branches. Focusing on the right-hand branch, one might conclude that being *strained* entails a kind of emotional distancing that being *on top of it* does not; but as careful attention to the left-hand branch reveals, this would be a mistake. The problem is analogous to the one of preserving conceptual intensions when transferring research results between contexts. In the present case, *recognition* becomes—quite undeservedly—the context within which the distinction made by *cognition* must be interpreted.

Third, *orthogonality* between properties—an important quality to which I shall return shortly—may come to suffer due to cognitive bias. In short, the researcher will, when forced to make first one distinction and then another, be less inclined to question the first than the second, regardless of which one is actually the most clear. If the second property provides a reason to respecify the first, the researcher might nevertheless fail to do so simply because the first makes sense at face value. It is all too easy, for instance, to fall into the trap of reading into being *recognized* a general positive feeling or some other quality that turns out to be incompatible with at least some instances of being *strained.*

### Wide Hierarchies

**Figure 2.**
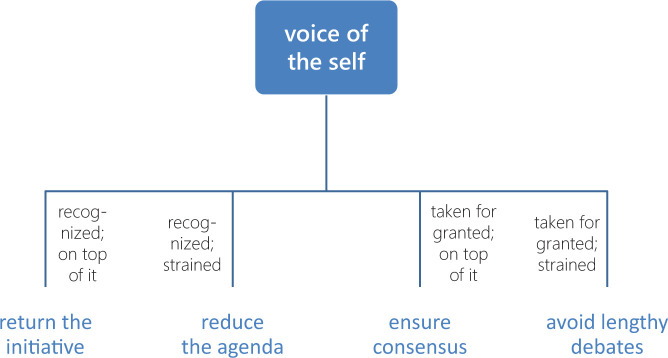
The four exemplary codes visualized in a wide hierarchy that gives equal weight to both properties *(recognition* and *cognition).* *Note.* Wide hierarchies gloss over differences and scale poorly.

In a *wide* hierarchy, at least some siblings differ with regard to more than one property (see Figure 2). Because no property is given precedence, wide hierarchies avoid at least some sources of bias. They do bring, however, at least two problems worth mentioning.

First, where deep hierarchies tend to falsely imply certain relational differences, wide hierarchies gloss over differences that do exist. Figure 2 does not, for instance, help one see that *ensure consensus* is more closely related to *return the initiative* than to *reduce the agenda*.

Second, wide hierarchies scale poorly. As subcategories grow in number, keeping track of their similarities and differences will grow increasingly difficult. Ultimately, a wide hierarchy threatens to degenerate into an amorphous mess that does little to help the researcher draw conclusions about causal connections.

To conclude, although conceptual hierarchies can be useful (at least initially) for organizing one’s thoughts, they are problematic because they steer the researcher toward mistaken conclusions about the structure of represented concepts. Regardless of one’s ontological assumptions, one clearly cannot consider conceptual hierarchies to be objective mappings of reality. Instead, they should be thought of as artifacts of tradition that can, despite best intentions, obfuscate rather than clarify conceptual relationships. Tools and procedures that emphasize hierarchies make dimensionalization cumbersome, sometimes necessitating workarounds such as creating separate hierarchies for dimensions (Hutchison et al., 2010, p. 290). Although such solutions might work after a fashion, they seem counterintuitive if the goal is to visualize the internal structure of a concept. Relying on rigid tools, computer-aided or otherwise, that enforce the use of a conceptual hierarchy is therefore a questionable practice that may blind the researcher to other possibilities.

## Principles of Multidimensional Property Supplementation

After these preliminaries, I will proceed to undertake the core task of this article, namely to propose a *multidimensional* approach to accounting for the relationships between the concept being studied and its codes. MPS rests on three principles that must be discussed in some detail before moving on to more practical matters. First, it is assumed that the concept can be fully characterized through a definition that declares its purpose in combination with properties that account for practically significant variability in its manifestations. Second, when variability is conceived of as taking place within a multidimensional space of possibilities, additional qualities emerge that aid theorization of the concept through explication. Third, because practically significant variability is often continuously distributed rather than categorical, an efficient account of the concept’s extension rests on maximally orthogonal, minimally skewed properties. In what follows, I will elaborate on each, hoping to show that they rest on a solid pragmatist foundation.

### Capturing the Concept’s Extension Through a Finite Set of Properties

To say that the extension of a concept can be *fully characterized* by some finite set of properties is to say that this set accounts for all practically significant variability. Obviously, no practicable set of properties could capture *every* conceivable difference between related phenomena, so the qualification “practically significant” is pivotal. The pragmatist truth criterion, according to which theory ought to be action-guiding (Corbin & Strauss, 2008, p. 2), entails selecting from multiple conceptual possibilities those that best illuminate, and hence best contribute to the solution of, the problem at hand. Wrote Peirce in 1878 about the concept of force,

The idea which the word force excites in our minds has no other function than to affect our actions, and these actions can have no reference to force otherwise than through its effects. Consequently, if we know what the effects of force are, we are acquainted with every fact which is implied in saying that force exists, and there is nothing more to know. (Peirce, 2011, p. 35)

Although qualitative researchers may balk at an example from physics, the case is not principally different in other fields. (The reader could try, at their leisure, deriving a theorem that makes more sense to them by substituting the name of their favorite concept for “force.”) The general implication of Peirce’s claim is that all we need to know about a concept can be formulated in terms of its observable effects in the world. The concept’s meaning thus consists of two parts. The first is its purpose: We think about force to explain (and successfully act upon) changes in motion. The second are the questions that must be answered with facts to enable us to make predictions: What matters in the case of force is magnitude and direction. Everything else that we might say about force can be reduced into its purpose and its properties.

In more general terms, a fully characterized concept is one that is, first, clearly enough defined to situate it firmly within the theory and making explicit what it is to explain or predict, and second, supplemented with a list of properties that help us look away from disturbing “noise” or “occasional contextual features” (Morse, 2004, p. 1390) so that we may focus on those differences that have bearing on other parts of our theory. The latter pursuit, which is my interest here, presents the researcher with a twofold challenge. First, whether a particular property is at all relevant depends on the aim of the theory, just as “[c]herry trees will be differently grouped by woodworkers, orchardists, artists, scientists and merry-makers” (Dewey, 2004, p. 88). Not much can be said in the abstract to aid such judgments. Second, the researcher must constantly navigate between *too little* and *too much* by making decisions of a different kind: whether to continue or stop looking, to make or overlook some distinction, to discard a property or to keep it. Judgments such as these are more promising targets for heuristics. In the following sections, I lay the foundation for MPS by expounding the following three heuristics for selecting properties:

1. Add properties until different codes are dimensionally distinguishable.
2. Employ parsimony to keep the concept grounded and simple.
3. Address redundancy through reduction.

#### Add properties until different codes are dimensionally distinguishable

According to the first heuristic, no two codes that differ in practically significant ways should be left to answer similarly the questions asked by their parent, for such a state of matters would indicate that some pertinent facts remain to be theorized. (In contrast, codes that do not differ significantly may safely co-exist; occasionally, they might even serve to inspire new thoughts and hypotheses.) To address untheorized differences between codes, the researcher adds properties until codes that are intuitively different are formally separated.

Consider an example from the study on the *voice of the self*. A recurrent theme was strain that threatened to overwhelm the GP’s cognitive capabilities. As I coded selectively, several codes came to suggest themselves as possible responses to such strain. One of them, *tie up loose ends*, was found to be too vague, subsuming incidents that were intuitively different. This led to the conception of a slightly different code, *reduce the agenda*, that could account for part of the incidents. To distinguish these two codes theoretically, a new property was then called for. Inspection revealed that *reduce the agenda* implied some degree of certainty without which a reductive move would entail a risk of missing something important, whereas *tie up loose ends* implied a degree of confusion by virtue of which the situation was already highly uncertain. The property that unified these contrasting dimensions was eventually named *degree of certainty*.

Properties, then, should never be added blindly; each must earn its place by *making a difference elsewhere*. This criterion saves us from the onus of adding properties forever, but also requires us to entertain already at this point some idea of the process that the concept will take part in.

#### Employ parsimony to keep the concept grounded and simple

A concept’s *efficiency* can be thought of as a function of its *explanatory power* and *simplicity*, both of which are arguably aspects of theory quality (Miller & Fredericks, 1999, p. 542; Soulliere et al., 2001, p. 256). Because we value efficiency, theorizing every practically significant difference is not always reasonable. There are two particular threats to watch out for.

One threat to efficiency is *sparsity*, which occurs whenever a combination of dimensions is unbacked by codes, and hence by data. It is often tempting to interpret sparsity as lack of theoretical saturation and to reflexively reach for the preferred remedy, theoretical sampling. Although theoretical sampling is indeed necessary to attain conceptual density (Becker, 1993, p. 256; Conlon et al., 2020, p. 948; Draucker et al., 2007, p. 1138), there are two caveats. First, if the properties that introduce sparsity interact, logically or empirically, in ways that preclude incidents manifesting certain combinations of dimensions, no amount of sampling will help. Second, sparsity limits the available material for making constant comparisons, without which one runs the risk of forcing data (Heath & Cowley, 2004, p. 144). While occasional gaps might be manageable by framing them as hypotheses and sampling theoretically, retracing one’s steps by removing some of the problematic properties is sometimes a safer course.

The other threat is rampant complexity. While high *resolution—*capacity for making fine distinctions—grants explanatory power to the model, it is also anathema to simplicity. Balancing these two concerns is a matter of identifying the point beyond which additional distinctions are conflatable with little loss in meaning, and then to apply Occam’s razor judiciously.

#### Address redundancy through reduction

Despite best efforts at parsimony, a potential problem may occur in the form of *redundancy* when one property includes another, or some aspect of it, in its definition. While theoretically possible with as few as two properties, redundancy becomes more of an issue as properties grow more numerous, particularly if insufficient attention is paid to how they interrelate. Like any dependency between properties, redundancy can entail sparsity: Given a redundant quality *R* such that dimension *A* implies *R* and dimension *B* implies *not-R*, sparsity occurs whenever one property declares *A* and another property declares *B*, for the case *A and B* is then contradictory. Barring coding idiosyncrasies, that particular combination of dimensions will then lack data to back it.

Redundancy differs from other kinds of dependency in that the gaps that it gives rise to will remain regardless of how much additional data are generated. This is because the cause is conceptual rather than empirical. To expose it, the researcher examines the properties and dimensions on a conceptual level, asking a series of questions such as the following:

- Are the properties clearly defined?
- Do the properties’ definitions entail their conceptual independence?
- Do the dimensions provide answers to the questions asked by their properties?
- Is each dimension compatible with every dimension of the concept’s other properties?

Only when all such questions can be answered in the affirmative has redundancy been ruled out. In the case that two properties are dependent in a way that puzzles the researcher, reducing them into components that can be more easily checked for synonymy might be helpful.

In principle, redundancy can be prevented by ensuring that all properties are irreducible. In practice however, strictly irreducible properties may not always be efficient, particularly if some of their dimensions are rarely seen. We are thus impelled to look for the “sweet spot” between irreducibility and efficiency. One way of conceptualizing it is in terms of *relative irreducibility*, beyond which further reduction would cause “loss of an epistemologically indispensable level of description and explanation” (De Sousa, 1990, p. 91). What levels of explanation are indispensable depends, of course, on the purpose of the concept, but as a general rule, the loss to be avoided can be conceptualized as *reduced practical utility from overcomplication*. Given, for instance, two irreducible, rarely seen, partially overlapping qualities *A* and *B* that have almost the same effects with no significant interactions, it might be more reasonable (and certainly more economical) to construct from them a compound that distinguishes between the two cases *A or B* and *neither A nor B.* The compound property is, on this understanding, relatively irreducible.

As we saw earlier, the *voice of the self* came to declare a dichotomous property named *cognition* with the dimensions *on top of it* and *strained.* This was a simplification, for cognitive strain is arguably a function of at least two variables—the complexity of the problem and the amount of available time—and the former could probably be reduced even further. However, as the data indicated that the effects of rushing through a moderately complex encounter were comparable to those of slightly hastening a highly complex one, distinguishing between these two cases was superfluous in the current context. After the relatively irreducible property *cognition* had passed the redundancy test, I was therefore satisfied with it. Had I instead insisted on completely avoiding reducibility, the model’s resolution might have outgrown its explanatory power.

### Explicating the Multidimensional Space of Possibilities

It is time to make explicit what I have only hinted at so far, namely that concepts can be interpreted as *multidimensional spaces of possibilities* in which all practically significant variability is (metaphorically speaking) physically separated. This metaphor not only aids visualization but also makes it clear that *relatively irreducible properties* grant us only a preliminary understanding of the concept’s extension. By considering how properties interact, the researcher can discover additional qualities which, although they cannot be deduced from their antecedents, nevertheless make sense in the light afforded by them. I refer to such a quality, given how it is “more than the sum of its parts” (Levers, 2013, p. 4), as *emergent.*

#### The spatial metaphor

A concept that has been defined in terms of its overall purpose and theorized through carefully selected properties has two important formal second-order qualities. First, given that redundancy has been avoided, its properties will be conceptually independent; they can thus be thought of as orthogonal *axes*. Second, the dimensions of any one property will be mutually exclusive; hence, they are *coordinates*. Taken together, these two qualities justify using the metaphor of *space*. Much like objects in physical space, codes that connect a concept to data can be thought of as occupying discrete positions in a space of conceptual possibilities. From this viewpoint, the spatial separability of codes is what marks them as theoretically distinct.

**Figure 3.**
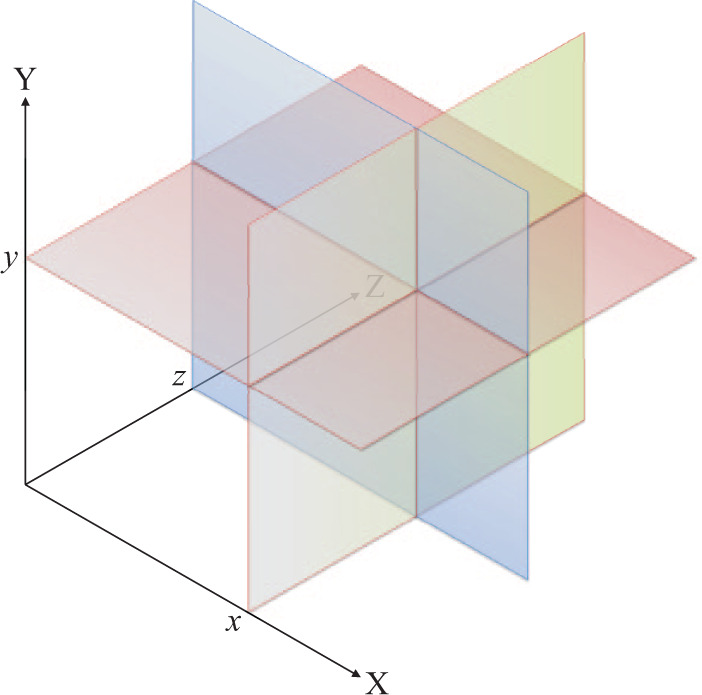
A 3-dimensional conceptual space is defined by three orthogonal axes. *Note.* Whenever a set of incidents manifest a specific coordinate (such as *x*, *y*, and *z* in this figure), every possible description of them will be restricted to the hyperplane perpendicular to the corresponding axis.

According to our spatial metaphor, the extension of a concept is an *n*-dimensional space defined by *n* axes (properties). Whenever a piece of data is assigned a coordinate (dimension), its vagueness is reduced because movement is no longer possible along the corresponding axis. Each coordinate thus corresponds to a *hyperplane* (an *n*–1-dimensional *subspace*) which is flat and perpendicular to the axis. Wherever two hyperplanes intersect, we find an *n*–2-dimensional subspace perpendicular to both. Compounding such intersections with additional hyperplanes yields subspaces of decreasing dimensionality, until we reach the 0-dimensional one, which is a *point.* As shown in Figure 3, flat hyperplanes in a three-dimensional space are planes.

A redundant yet convenient term is the *codimension* of a subspace, which is the number of coordinates that define it. For instance, within a 3-dimensional space, a 1-dimensional subspace—one that allows variation along a single axis—is defined by two coordinates; hence, it is 2-codimensional. Finally, representations of the conceptual space that take into account variation along less than the full number of axes are *projections*. A 2-dimensional projection, for instance, is constituted by two axes, regardless of the dimension of the ambient space. In the simplest possible 2-dimensional projection, both properties are dichotomous.

As an example, in the 4-dimensional conceptual space of the *voice of the self*, I sought the meaning of an intersection between three hyperplanes: *unsuited, strained*, and *taken for granted*. By considering its position in three 2-dimensional projections, I could also describe the resulting 3-codimensional subspace as a combination of being *oppressed, cornered*, and *useless*. This granted me an intuitively appealing explanation to why every incident in this subspace appeared to be, in some way or another, about *regaining power*. The subspace was 1-dimensional in that it still allowed variation along the fourth property, *degree of certainty*.

What this brief mathematical excursion grants us is the ability to define *conceptual subspace* as *a subset of the concept’s extension, demarcated by coordinates (dimensions) along one or more axes (properties) that are declared or inherited by the concept.*

#### Inferring emergent qualities of subspaces

The dimensions that define a subspace constitute it in the sense that dimensions cannot be added, redefined, or removed without altering its meaning. But the meaning of a subspace is not exhausted by the meanings of its dimensions. On the contrary, it should be possible to add, through repeated observation, practically significant facts to the definition of subspace *A and B* beyond what can be deduced from the definitions of *A* and *B* alone. In that case, the subspace in question has an emergent—unpredictable, or transcendent (Levers, 2013, p. 4)—quality.

**Figure 4.**
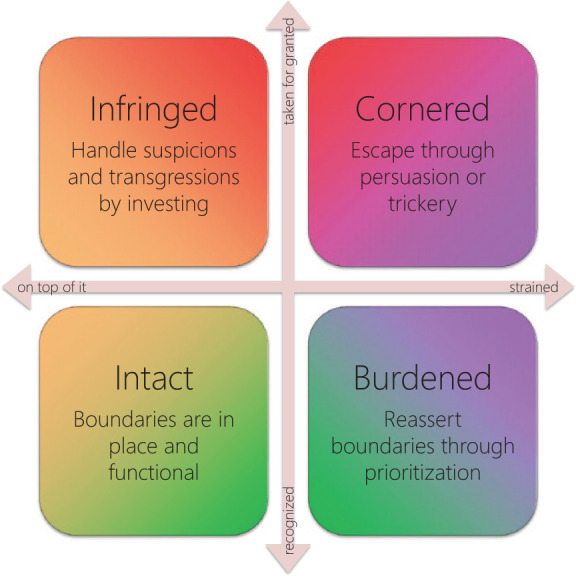
A 2-dimensional projection of the *voice of the self* in which properties *cognition* and *recognition* act as orthogonal axes reveals emergent qualities of the 2-codimensional subspaces. *Note.* These emergent qualities are unified by their relationship to the GP’s *integrity.*

For example, one of the 2-codimensional subspaces within the *voice of the self* came to represent the experience of being *cornered* (see Figure 4). This happened when the GP was both *strained* (being exposed to too much data, tasks and disturbances within a given time frame) and *taken for granted* (being expected to deliver predefined products rather than to exercise professional judgment). In these situations, setting one’s priorities would incur sanctions or disdain, whereas going along with expectations would aggravate the strain. Quite understandably, one emergent quality of *cornered* was that the GP, expecting their usual strategies for handling the component threats to do poorly, sought to escape the situation by persuasion or trickery. Another emergent quality, which applied to the complementary subspaces as well (that is to say, to the whole 2-dimensional projection), was that the properties of cognition and recognition both implied the possibility of someone or something *intruding* on the GP, thus implying a threat to *integrity*. Neither emergent quality could be deduced from the definitions of cognition and recognition alone; it was only after thinking about the subspaces and comparing their subsumed codes that such conclusions could be drawn.

To include emergent qualities in the definition of a subspace is to *explicate* it, improving upon its meaning by supplementing it (Quine, 1980, p. 25). Explication has the effect of making the subspace more convincing by itself and less dependent on purported examples, hence less vague in the sense suggested by Peirce (2011, p. 295). The other side of the coin is that the projection is rendered susceptible to falsification by examples that challenge its comprehensiveness, that is, cases that do not match any of its subspaces. As an example, I considered at one point *trust* as a property of the *voice of the self*, only to find that it introduced a false dichotomy between being trusted and distrusted that did not capture prevalent cases of *indifference.* This prompted a respecification of the concept that led up to the conception of another property, *recognition*.

Because emergent qualities can be explained by, but not analyzed in terms of, lower-level qualities (De Sousa, 1990, p. 32), explications of subspaces will always make reference to facts beyond those represented by the constituting dimensions. As an example, the experience of being cornered is emergent from the dimensions of being *strained* and *taken for granted*, just like the aesthetic features of a painting are emergent from how it reflects light; in neither case can the matter of *that* being experienced *like this* be deduced from the lower-order qualities of *that*. Once the connection has been pointed out however, it should be a simple matter to agree to it. Determining whether an observed pattern indicates a conceptual or causal relationship is a sometimes difficult task (Soulliere et al., 2001, p. 267) where explication can be helpful, for in the contrasting case that the emergent qualities make no sense in the light of their constituent dimensions, one may have stumbled upon *incommensurability—*the state of matters where one or several of the properties in question belong, if at all relevant, elsewhere in the theory.

### Maximizing Efficiency by Considering the Distribution of Data

I have so far argued that a parsimonious selection of independent properties can make codes dimensionally distinguishable and that such properties can be combined to produce subspaces with emergent qualities. In what follows I hope to show that whenever practically significant variability is continuously distributed rather than categorical by nature, we have reason to make these properties maximally orthogonal and minimally skewed, lest the model become wasteful. We shall see how complementing our qualitative attention to words and meanings with quantification of events can yield useful insights without committing us to the arguably “false assumption that frequency implies importance” (Bringer et al., 2006, p. 254) or otherwise transgressing methodological boundaries (Wilson & Hutchinson, 1996).

**Figure 5.**
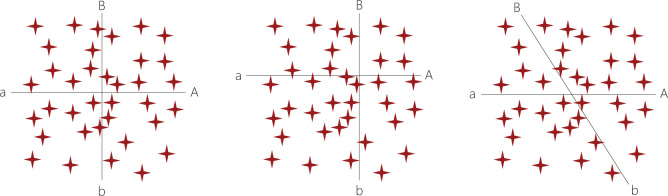
Two dichotomous properties that partition the data into four subspaces can be orthogonal and non-skewed (left), skewed (middle), or non-orthogonal (right). *Note.* A combination of non-orthogonality and skew is also possible (not shown).

The following thought experiments presume two hypothetical properties *Aa* and *Bb*, both of which dichotomize naturally continuous data. Consider first the ideal case where *Aa* and *Bb* are orthogonal and minimally skewed (see Figure 5, left). Unlike multimodal distributions which lend themselves quite naturally to categorization, unimodal or uniform distributions allow no cutoff that is not at least somewhat arbitrary. However, as long as the model distinguishes paradigmatic cases and is fairly efficient overall, this is acceptable. While it does impose a *formal* structure on the data by applying a general heuristic, this should not be confused with the problematic practice of forcing data into compliance with a preconceived falsifiable hypothesis (Heath & Cowley, 2004, p. 143; Kelle, 2007, p. 149; Melia, 1996, p. 373).

#### Detecting skew as sparsity

Depending on how the researcher defines the dimensions of a property, it might come to suffer from *skew* (see Figure 5, middle). A skewed property is one that fails to partition the conceptual space into equally sized subspaces (where “size” is the unconditioned probability of containing a randomly sampled data point). Although based on the same raw data as the previous diagram, this one is markedly different. In particular, because both properties are skewed, subspace *ab* contains the majority of all data points, whereas subspace *AB* is sparsely populated.

Three aspects of skewness are worthy of note. First, a skewed property does not imply real-world skew; rather, skew (or its absence) reflects how we conceptualize the phenomenon of interest, including our choice of dimensional cutoffs. Second, skew incurs a loss of resolution that could impede modeling of cause–effect relationships. In the diagram, our inability to distinguish between the numerous cases within subspace *ab* is potentially problematic, as is the sparsity within subspace *AB*. Third, in a realistic setting, our only clue to the presence of skew is an unequal distribution of data points between subspaces. While skew can be detected by considering properties in isolation, a multidimensional approach provides a more sensitive test because the effects of individual skews are multiplied.

Because we value conceptual resolution, avoiding skew is—all else equal—a worthy goal. It is also a mostly attainable one, except in the case of categorical or multimodal distributions.

#### Detecting non-orthogonality as empirical dependence

The two diagrams discussed so far show no signs of significant *non-orthogonality*, which I shall define as *an empirical correlation between properties to the effect that variation along one predicts variation along another*, for in neither case does knowing that some hypothetical data point manifests dimension *A* help us predict whether it also manifests dimension *B*, or vice versa. The third diagram presents a contrasting case (see Figure 5, right). Notably, because equally many data points manifest dimensions *A, a, B*, and *b*, neither property is skewed. What sets this case apart is that some conditional probabilities differ from the corresponding unconditional ones. If we know, for instance, that a data point manifests *A*, we also know that it will be more likely to manifest *B.* That such predictions are possible indicates that the properties are non-orthogonal. Although I have drawn the *Bb* axis obliquely to illustrate the point, a real study will of course lack such giveaways. The researcher might therefore need to pay attention to relative frequencies, and perhaps even use some basic statistical tests to check for non-orthogonality.

There are several possible causes of non-orthogonality. As always, selection bias is one. A second, idiosyncratic coding, is particularly likely before codes have been clearly defined. A third is the presence of causal connections between properties, either directly or through confounders or mediators. While non-orthogonality may resemble redundancy, it is an empirical matter, hence our methods for revealing it are very much data-driven; a conceptual subspace might be logically consistent yet empty, indicating that the constituting combination of dimensions is perfectly possible yet unlikely to be seen for empirical (for instance, contextual) reasons. Non-orthogonality does not necessarily pose a serious problem, but is better thought of as a source of relative inefficiency, and sometimes one of lack of theoretical saturation.

The upshot of this venture into the realm of pseudo-quantification—which many qualitative researchers will no doubt find off-putting, but which I believe is in line with the original intent of grounded theory as a general methodology (Glaser, 1999, p. 842)—is this. Whenever the data are not naturally categorical, the researcher has good reason to explore signs of non-orthogonality or skew. To detect some sources of inefficiency, they need to pay at least passing attention to frequencies. While skew can in principle be identified by considering properties one by one, a multidimensional approach is clearly more sensitive. Non-orthogonality, being a multidimensional phenomenon, cannot be exposed without a multidimensional approach.

## MPS as an Iterative Process

Having thus established the theoretical foundation and basic principles of MPS, I will now describe how the method is applied in practice to a would-be concept—a category—within a grounded theory study. In an iterative process, codes and properties are added, tested, and removed using Gerson’s (1991) “heterogeneity supplementation heuristics” until they account for variation within the category’s extension. The product is a multidimensional model that characterizes the category—now a concept—through relatively irreducible properties and dimensions as well as subspaces that carry emergent qualities.

### The Four Steps of Supplementation

Supplementation is a process through which material for one’s theory is expanded and organized:

Conceptually, it lies between coding (which names categories and specifies the properties associated with them), and theoretical sampling (which tells us what kinds of site or situation we want to look at next). Supplementation starts with an extant category, and systematically elaborates contrasting categories in order to provide the “raw material” for theoretical sampling, cross-cutting and densifying theories, and testing hypotheses. The focus of supplementation is thus on categories, not on data; on “might be” rather than “is.” (Gerson, 1991, p. 2)

Gerson distinguishes three classes of heuristics which he names *differentiation, reallocation*, and *homogenization*. Differentiation “goes from one thing to many things,” increasing the heterogeneity of the material. One of its uses is to discover conceptual siblings that are similar to or different from some category. Reallocation “goes between many similar and many dissimilar things” whereby one’s focus is shifted from what the constructs have in common to how they differ, or vice versa. Finally, homogenization “goes from many things to a single thing,” decreasing the heterogeneity of the material, for instance, by creating a category that subsumes existing codes or subcategories.

Gerson also hints at the possibility of supplementing properties, a process that he regards the “mirror image” of supplementing categories:

When we use the processes of differentiation, reallocation and homogenization to supplement categories, we refer (tacitly or explicitly) to some criterion property to frame the boundaries of “similar” and “different.” When we use the same processes to supplement properties, we must use some criterion category to frame the boundaries of “similar” and “different” in the same way. (Gerson, 1991, p. 14)

**Figure 6.**
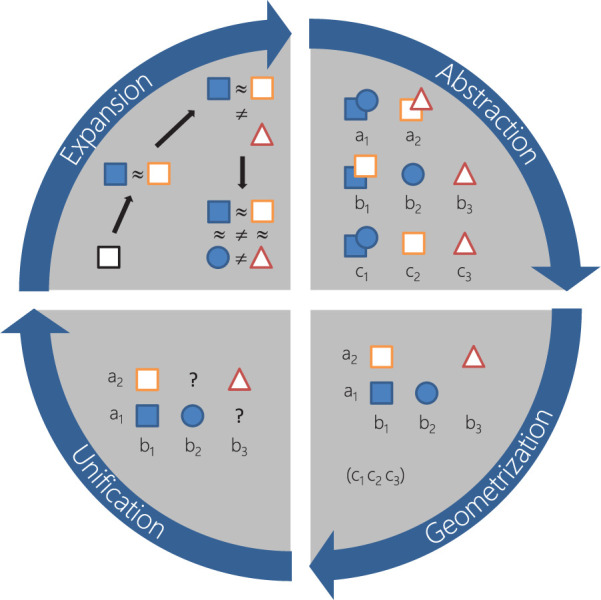
The four steps of supplementation: splitting vague codes and hypothesizing contraries (*expansion*), framing differences in terms of properties and dimensions (*abstraction*), defining subspaces that account for interactions between properties (*geometrization*), and validating the concept against data and disposing of constructs irrelevant to theory (*unification*).

I will presently describe how supplementation of properties can be used in practice to elaborate a concept multidimensionally, drawing upon examples from the study on the *voice of the self*. The astute reader will notice that two of the steps involve hypothesis testing which, as argued by Gerson, is not itself a matter of supplementation but rather of theoretical sampling. In MPS however, testing is inextricably linked to supplementation because the process presumes a simultaneous but phase-shifted supplementation of codes (see Figure 6).

#### Expansion (code differentiation—property testing)

In the first step, the researcher expands the code base by making distinctions beyond those that the current model predicts. Expansion comes in two forms: *splitting* codes that appear vague, and *overturning* those that lack clear contraries.

A code is *split* by being replaced by more precise ones. Because splitting is intuitively rather than theoretically driven, a property that captures the distinction will often be lacking. As an example, the code *reduce the agenda*, representing the imperative to reduce the size of one’s to-do list to alleviate pressure, seemed heterogeneous in ways that could not yet be theorized. It was therefore split into two codes, *turn a blind eye* and *set the agenda*, both of which retained some of the meaning of the original code while being more precise.

To *overturn* a code is to use the “flip-flop” technique (Corbin & Strauss, 2008, p. 79) to generate a contrary one, using the concept as the frame that connects them and explains what it means for them to be “different.” For instance, the code *return the initiative* came over time to represent the imperative to reduce one’s burden by taking a step back—into the role of consultant, as it were—while leaving the other (the patient or co-worker) in charge of the problem. To complement it, I hypothesized the imperative *stay in control* which might also serve to protect the self in sufficiently different circumstances.

Besides adding codes that potentially increase the theoretical resolution of the concept, this step tests the hypothesis that the current set of properties suffice to make all necessary distinctions.

#### Abstraction (code reallocation—property differentiation)

To make the concept “applicable to many similar situations and contexts,” the researcher carries out “the analytic work of identifying attributes, moving beyond emic tag labels and developing careful definitions,” which Morse refers to as “decontextualization” (Morse, 2004, p. 1390). I prefer the term *abstraction*—extracting from “detached observations certain general characters in which the observed phenomena resemble one another” (Mill, 1884, p. 376)—because the purpose is to make the concept theoretically useful, whereas any loss of context is merely incidental. In MPS, the researcher abstracts by reallocating codes, framing their similarities and differences—so far only intuitively understood—in terms of properties and dimensions which are simultaneously differentiated. Depending on how similar or different the codes in question are, the procedure becomes predominantly one of *separation* or *consolidation*.

*Separation* is the theorizing of differences between mostly similar, but intuitively distinct, codes. One approach is to list dimensions that could be attributed to one code but not the other, select those that best bring out the difference, and create a property that pins down the disagreement. Recall the two codes *set the agenda* and *turn a blind eye* that emerged through *splitting* in the previous step. Closer inspection revealed that when GPs *set the agenda*, they felt that they were the right person to handle the problem—in effect, *making a difference*—whereas *turn a blind eye* indicated the feeling of being *unsuited*, with the implication that their skills could be put to better use elsewhere. To unify these dimensions, I devised the property *purpose*, which came to stand for variations along the range of being or not being in the right place.

*Consolidation* is about identifying the common denominator that unifies contrary codes. As there will be no shortage of candidate dimensions to mark them as different, the challenge is rather to identify the most crucial properties on which they “agree to disagree.” To this end, it can be effective to ask “In what regard do these codes stand for opposite things within the frame provided by the concept?” This allowed me to discover that the code *return the initiative* implied a greater degree of *certainty*, or capability of making predictions, than did *stay in control*.

#### Geometrization (code homogenization—property reallocation)

In this step, supplementation is driven by *selection* and *juxtaposition* of properties. Through these procedures, properties are reallocated to create subspaces of varying codimensionality.

Property *selection* involves grouping similar properties and selecting from each such group the property that best captures a practically significant difference. Good candidates are generally those that are relatively irreducible, lack skewness, and tie in with other concepts. As an example, the property *purpose* was chosen over *responsibility* because it was less reducible and showed no signs of redundancy with other properties. The property *recognition* turned out to be superior to *social acceptance* for similar reasons.

For a set of selected properties to be good all things considered, the implied conceptual space must be efficient. To this end, the researcher *juxtaposes* properties in a series of 2-dimensional projections and examines the codes subsumed in each of the resulting 2-codimensional subspaces. Each subspace is named and explicated, whereby emergent qualities are drawn out that add to—but are coherent with—the meanings of the constituting dimensions. If the properties are commensurable, the researcher should be able to name the projection as well. The same procedure is applied on each level of codimensionality until all practically significant properties (and all resulting *n*-codimensional subspaces) are considered simultaneously.

Property reallocation is accompanied by code homogenization, which sees codes subsumed under conceptual subspaces, thus reducing their code base footprint and theoretical relevance. In return, theoretical complexity increases with the proliferation of subspaces.

#### Unification (code testing—property homogenization)

Working with a narrow set of abstractions incurs a risk of losing contact with both data and other parts of the theory. In this final step of the supplementation cycle, the researcher therefore employs *specification* and *contextualization* to reduce the complexity of the concept and situate it firmly in data and context.

**Figure 7.**
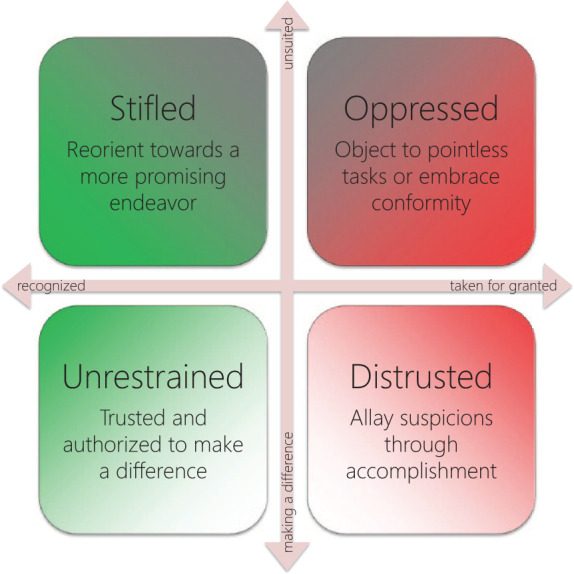
Geometrizing dichotomous properties *purpose* and *recognition* reveals the *autonomy* projection. *Note.* Because it intersects the *integrity* projection, being *oppressed* or *distrusted* implies being either *cornered* or *infringed*, and vice versa.

*Specification* involves revisiting codes and associated data and memos while paying attention to how codes reflect data in the light of the now slightly better characterized concept. Ensuring “fit” between codes and their containing subspaces is crucial to overcoming the concept-indicator problem (Layder, 1998, p. 79). As an example, in a projection juxtaposing the properties *purpose* and *recognition* (see Figure 7), the code *refuse to clean up other people’s messes*, signifying the GP’s exasperation at being implicitly assigned menial tasks, ended up in a subspace named *oppressed.* This subspace came to subsume instances where GPs were sidetracked from working toward valued goals and insufficiently respected to have their concerns heard unless they raised their voice. This drew my attention to the fact that autonomy can be infringed not only directly (through directives and threats of sanctions) but also indirectly (through negligence or indifference). Because its position in the other projections implied less severe component threats to the self, I inferred that *refuse to clean up other people’s messes* might be more about fighting for one’s autonomy than about, say, reducing one’s workload. By revisiting the backing data I could confirm this, and also found evidence supporting a number of corollary hypotheses, for instance, that being expected to clean up after others implied an *infringement* of integrity.

In parallel, properties are homogenized by discarding those that fail to produce subspaces significant enough to warrant the increased complexity. Given that those that remain tie in strongly with other parts of the theory (as they should), this is a process of *contextualization* that can be applied to any concept regardless of its role in the emerging theory. For instance, conditions can be related to consequences that reinforce or challenge them using the same techniques as when interactions are contextualized through a theoretical account of causal and intervening conditions. Indeed, until a core category has been identified, it may not be at all obvious which categories represent the theory’s context (Scott, 2004, p. 121).

I defined the experience of being *cornered* as follows:

The GP works under the watchful eye of others, and the situation is too complex to be grasped within the allotted time. As the GP cannot simultaneously appease others and establish boundaries against the torrent of data and tasks, they are cornered, and their integrity seriously compromised. To escape, the GP must somehow convince others to cut them some slack.

This is clearly not a dictionary definition of the word “cornered,” but an explication that emerges from specific observations in a specific context. It goes well beyond the logical antecedents summarized in its first sentence and ties in—despite being abstract—with the context, embedding several factual claims that make reference to other parts of the theory: that the GP at least occasionally *interacts* with suspicious others; that the *conditions* surrounding such interactions may be cognitively complex; and that two potential *consequences* are gaining slack or losing integrity. Whether the definition contextualizes enough can be debated, but it clearly aspires to do so. Crucially, there is no trace of specification which, while procedurally important, would encumber the theory if allowed to linger in definitions.

In the unification step, any sparsity that indicates gaps in the concept’s empirical support is addressed. In the case of redundancy, skewness, or non-orthogonality, simplifying or modifying the model may be advisable. Barring theoretical causes, there are also procedural ones to consider, such as lack of theoretical saturation and coding idiosyncrasies. Before remedying such issues through theoretical sampling and recoding, respectively, the gaps should be reformulated as hypotheses.

## Discussion

Despite being an acclaimed and widely used methodology for theory building, grounded theory continues to raise ontological, epistemological, and methodological questions. In the face of the evolution and proliferation of grounded theory methodologies (Annells, 1996; Hallberg, 2006; Melia, 1996; Rieger, 2019; Robrecht, 1995), it is fair to ask not merely what kind of methodological problems MPS can be helpful in solving, but also how compatible it is with various competing paradigms. After arguing that MPS fits well within grounded theory from the point of view of ontology, epistemology and methodology, I turn to the particular methodological strengths and weaknesses of the method. I conclude this article by outlining some possible uses of the method in the grand scheme of things.

### Ontological, Epistemological, and Methodological Concerns

Whereas some methodologists consider ontology methodologically relevant (Annells, 1996, p. 379), others opt to exclude such questions from consideration because “[r]esearchers generally treat social concepts as if they are real enough to be named, investigated, and analyzed” (Carter & Little, 2007, p. 1326). I am sympathetic to the latter view because it embodies the classical pragmatist idea that “real” amounts to “being as it is regardless of what you or I may think about it” (Peirce, 2011, p. 265), which opens the door to fallibilism (Hookway, 2016) without forcing us to either assume that our beliefs somehow “represent” reality or seek refuge in relativism. From this point of view, the debate on whether knowledge is “discovered” or “created” (Levers, 2013, p. 4) appears to have little practical import.

In contrast, the view that *epistemological* assumptions restrain methodology is more widely held. Many of the restraints in question concern matters of design and procedure on which MPS is silent: “which research questions we ask, the data we collect, our relationships with research participants, and how we render our analyses” (Charmaz, 2017, p. 4). As for epistemological positions that are clearly incompatible with MPS, for instance the preference of predefined, sequentially applied methods (Carter & Little, 2007, p. 1321), those seem unlikely to be held by grounded theorists. To embrace a pragmatist epistemology, as many grounded theorists do, is to hold that the purpose of empirical inquiry is to provide justification for our beliefs (Avis, 2003, p. 997). What matters from this point of view is that the theory and its concepts *work* (by explaining or predicting the phenomenon of interest) while remaining amenable to rejection or modification whenever they clash with experience (Quine, 1980, p. 42).

Methodologically, MPS does challenge some aspects of grounded theory, albeit on a rather technical level, through its questioning of traditional conceptual hierarchies. This might already mark it as suspect in the eyes of some readers, for methodology certainly provides a method with a direct mode of justification (Carter & Little, 2007, p. 1326) that MPS must do without. That said, it is questionable whether methodological justification is at all to be coveted, as it relativizes beforehand the method’s output to a particular conceptual scheme (Avis, 2003, p. 996). Arguably, claims to knowledge “cannot be reduced to a demonstration that the evidence has been generated through the application of rules and procedures derived from a coherent methodological theory” because this presumes a degree of acceptance of techniques that cannot be taken for granted outside the research tradition (Avis, 2003, p. 1003). Following this train of thought, I believe that critique of any grounded theory method should be funneled by the realization that grounded theory must, to be credible to a wider audience, be at least somewhat open to modification and extension.

### Strengths and Weaknesses

This method is not intended to be applied indiscriminately to every concept across a theory. Researchers will find it useful, I believe, for elaborating complex concepts: pivotal points in addressing the main concern; choices between mutually exclusive strategies; or closely related conditions that, through their interactions, give rise to effects that are more than the sum of their parts. I would caution against using it to lump together unrelated phenomena or ones that can only be shoehorned together on the grounds that they are all “conditions” or “strategies.” Failure to name the concept accurately and evocatively is a bad sign in this respect.

One advantage of multidimensional conceptual models over hierarchical ones is that they can be made more robust, seeing as subspace definitions are literally triangulated from those of the ambient concept, constituting properties and dimensions, and subsumed codes. This is not to say that MPS is purely accommodationist (Miller & Fredericks, 1999, p. 546). Because its emergent qualities predict features of events, a multidimensional conceptual model is a falsifiable hypothesis in its own right. A conceptual model that can be falsified or corroborated also through non–grounded theory methods would seem to be a promising basis for the kind of *epistemological* justification that some authors seek (Avis, 2003, p. 1003). On the other hand, as multidimensional conceptual models grow more complex, they can become cognitively challenging. As the number of subspaces increases exponentially with the number of properties and dimensions, one must consider each addition carefully, be prepared to remove inefficient properties, and construct composites of properties that make meaningful distinctions together but not quite individually. To keep the model simple, I strongly advocate dichotomous properties over finer-grained ones, except where the distribution appears to be multimodal.

A crucial consideration for the purposes of this article regards the relationship between methods for concept development on one hand and theoretical sensitivity and openness on the other. It is not immediately evident that grounded theory methods in general are conducive of theoretical sensitivity, or the ability to “see relevant data” (Kelle, 2007, p. 136); it has been argued to the contrary that preoccupation with technically complex methods tends to overshadow other considerations (Thorne, 2011, p. 446), divert the researcher’s attention from the data (Kools et al., 1996, p. 315; Robrecht, 1995, p. 171), distract from actual theory building (Melia, 1996, p. 376), and be “counterproductive to the spirit of creativity” (Wilson & Hutchinson, 1996). Furthermore, method descriptions often give the false impression that the researcher is to follow a strict—or even rigid—sequence (Carter & Little, 2007, p. 1317; Layder, 1998, p. 28), whereas actual research practice may be considerably messier. Junior researchers in particular should be cautioned not to focus on the procedural aspects of the method to the detriment of insight.

One final concern regards the process of dimensionalization which, although a necessary step in the evolution of a category into a concept (Morse, 2004, p. 1390), incurs at least some risk of data forcing (Walker & Myrick, 2006, p. 552). Although the insights that “data never speak for themselves” and that interpretation “always requires moving somewhere” (Sandelowski, 2010, p. 79) may offer some consolation, one should be aware that interpretation is always relative to some frame of reference or another, be it preconceived or emerging. For this reason, it matters greatly what knowledge the researcher draws upon when choosing their direction of inquiry. The necessary practice of discarding theoretical constructs that are not “part of the world being investigated” (Cutcliffe, 2000, p. 1480) can be facilitated by postponing MPS until the concept has been located in one or several processes, at which point judging which of its properties actually make a difference elsewhere will be more straightforward.

### The Use of Induction in Explicating Subspaces

Miller and Fredericks (1999) have argued that the focus of grounded theory on theory development has come at the cost of attention to “the more specific mechanisms of a logic of discovery” and that an account of how grounded theories actually explain is therefore lacking (p. 549). In what follows, I will argue that MPS adds, if not “specific rules of inductive inference,” then at least heuristics that draw upon such rules.

Somewhat confusingly, “induction” is sometimes used to denote the mode of inference that Peirce referred to as *abduction, retroduction*, or *hypothesis* (Kelle, 2007, p. 144; Peirce, 2011, p. 151), the aim of which is to explain a surprising or puzzling observation. Induction in a narrow sense—the creation of generalized knowledge through repeated observation—takes place in MPS whenever subspaces are explicated. Consider the following passage in Mill’s account of the methods of agreement and difference:

The Method of Agreement stands on the ground that whatever can be eliminated, is not connected with the phenomenon by any law. The Method of Difference has for its foundation, that whatever can not be eliminated, is connected with the phenomenon by a law. (Mill, 1884, p. 484)

In MPS, the method of agreement is used to eliminate properties that are irrelevant to an emergent quality. Given an hypothesized subspace–quality relationship, the researcher looks for *cases* (that is, incidents that manifest the same quality) in other subspaces than the focal one. Whenever cases are found along the range of an axis (property), agreement has been shown to be lacking; that property can then be eliminated as irrelevant to the quality. As an example, the code *set the agenda*, residing in the intersection between *making a difference, certainty, recognized*, and *strained*, appeared to manifest an emergent quality of *prioritization*. By revisiting the data however, I found cases of prioritization where the GP was not *making a difference*, but rather felt *unsuited.* Consequently, the property *purpose* could be eliminated from consideration.

The method of difference is used to identify properties that are relevant to an emergent quality. Setting out from the highest-codimensional subspace that contains the known cases, the researcher looks for non-cases in subspaces adjacent to it on any axis. If a subspace is found that contains only non-cases, the axis (property) that joins the two subspaces together can be deemed relevant. (If a subspace contains both cases and non-cases, the emergent quality may be incongruent with how the dimensions have been defined, or else a property is missing that could make the necessary distinction.) As an example, compared with the 3-codimensional subspace from which *prioritization* was hypothesized to emerge, subspaces adjacent to it told rather different stories about *asserting territory, deepening the analysis*, and *being respected.* As these were all non-cases, all three constituting properties could be confirmed as relevant, thus corroborating the hypothesis.

### Multidimensional Concepts in Process

The relationship between MPS and methods for theorizing process is bilateral. We have already seen how causal hypotheses are used during the unification step to homogenize properties. The method’s *raison d’être*, however, is its propensity to produce nodes that help the researcher take the sometimes difficult step out of axial coding into actually developing theory (Kendall, 1999, p. 755). For this purpose, a good typology is one that aids theoretical elaboration by suggesting connections between emergent concepts (Layder, 1998, p. 73). In MPS, there are two options, each of which has its merits.

#### Subspace-based nodes facilitate abductive inference

Causal hypotheses may appear spontaneously in the guise of emergent qualities of subspaces that echo other parts of the theory. Such overlaps being ripe targets for theorization, a reasonable next step is to investigate the extensions of the involved subspaces—the one currently under scrutiny, and the one that it resembles—while applying abductive inference (Conlon et al., 2020, p. 948) to spell out possible horizontal (temporal or causal) relationships between them. Testing such hypotheses against theoretically sampled data can provide grounds for either rejection or corroboration (Dewey, 2004, p. 89; Heath & Cowley, 2004, p. 144). In the case of the latter, the theory can now be enriched with another inductively derived piece of generalized knowledge.

For instance, it appeared that when GPs were *pragmatic* about their professional ethics, making use of their profession as a tool rather than tending to it as an end in itself, they were also likely to be, in the language of the *voice of the self, certain* yet struggling to *regain power*. I therefore hypothesized that such a message from the *voice of the self* would cause the pragmatic stance (given certain conditions that were not yet fully understood). Repeated observation confirmed that within the implied 4-codimensional subspace—which was eventually named *being exploited*—GPs almost always acted pragmatically. A generalized conclusion could now be formulated: When an exploited GP holds superior knowledge as their sole asset, they tend to use it for seizing back power rather than upholding professional ideals.

#### Property-based nodes facilitate parsimonious causal claims

If traditional conceptual hierarchies are blunt tools for understanding vertical conceptual relationships, they fare no better with horizontal ones. Whenever one subcategory differs from another in more than one respect, causal hypotheses that draw upon their differences will easily come to overstate the relevance of merely contingent qualities. One might reasonably worry that conceptual subspaces will lead back to this quandary. They have, however, the redeeming qualities of being explicit about their constituting dimensions and—purportedly—mutually exclusive and together comprehensive with regard to conceptual possibilities. This makes it possible to rework hypotheses that reference subspaces into ones that test the respective contributions of the constituting properties. This logico-deductive approach, which resembles how MacFarlane and O’Reilly-de Brún (2012) estimated likelihoods of an ideal outcome given certain conditions, makes for more parsimonious causal claims.

As an example, although it was obvious at an early stage that the *voice of the self* tended to drive GPs toward self-protection, an effect that was expected to be more pronounced the greater the perceived threat to the self (Johnsson & Nordgren, 2019), the precise nature of this relationship eluded me until I had elaborated the concept. An early insight was that GPs tended to act in their own interests when they perceived their labor to be *useless* rather than *effective*. Far from being content with this finding, I dug down into the relatively irreducible properties that defined these subspaces, namely *purpose* and *cognition*. I then found that the effect was mediated by the former through the experience of being *unsuited*, whereas the implied association with being *strained* was spurious. Without a multidimensional approach, I might have come to draw false conclusions about these relationships, especially because they were not deterministic.

## Conclusion

A grounded theory concept can be construed as a multidimensional space of possibilities in which the concept’s properties take on the roles of orthogonal axes. I have here presented an iterative method for supplementing properties which is attentive to variation within data and facilitates theoretical separation of intuitively different events into subspaces, each of which is constituted by a unique combination of dimensions. I have argued that the method, wherever it departs from established methodology, is epistemologically well-founded because its basic principles—full characterization through properties, drawing out emergent qualities through explication, and efficiency through attention to frequencies—all adhere to the tenets of pragmatism. Using concrete examples from my research, I have demonstrated how the method fits within grounded theory methodology, and how some of its heuristics draw upon Mill’s rules of inductive inference. By virtue of being based on independent, relatively irreducible properties, multidimensional conceptual models are robust against several kinds of bias that plague conceptual hierarchies. A fully developed multidimensional concept can be easily connected to others in a process. All in all, I believe that multidimensional property supplementation is a worthy addition to the grounded theorist’s arsenal of methods for analysis and theorization.
